# Myc-binding protein orthologue interacts with AKAP240 in the central pair apparatus of the *Chlamydomonas* flagella

**DOI:** 10.1186/s12860-016-0103-y

**Published:** 2016-06-10

**Authors:** Venkatramanan G. Rao, Ruhi B. Sarafdar, Twinkle S. Chowdhury, Priyanka Sivadas, Pinfen Yang, Prabhakar M. Dongre, Jacinta S. D’Souza

**Affiliations:** UM-DAE Centre for Excellence in Basic Sciences, Kalina campus, Santacruz (E), Mumbai, 400098 India; Wehr Life Sciences, Marquette University, P.O. Box 1881, Milwaukee, WI 53201-1881 USA; Department of Biophysics, University of Mumbai, Kalina campus, Santacruz (E), Mumbai, 400098 India

**Keywords:** *Chlamydomonas reinhardtii*, MYCBP-1, A-kinase anchoring proteins (AKAPs), Flagella, Central pair, FAP174

## Abstract

**Background:**

Flagella and cilia are fine thread-like organelles protruding from cells that harbour them. The typical ‘9 + 2’ cilia confer motility on these cells. Although the mechanistic details of motility remain elusive, the dynein-driven motility is regulated by various kinases and phosphatases. A-kinase anchoring proteins (AKAPs) are scaffolds that bind to a variety of such proteins. Usually, they are known to possess a dedicated domain that *in vitro* interacts with the regulatory subunits (RI and RII) present in the cAMP-dependent protein kinase (PKA) holoenzyme. These subunits conventionally harbour contiguous stretches of a.a. residues that reveal the presence of the Dimerization Docking (D/D) domain, Catalytic interface domain and cAMP-Binding domain. The *Chlamydomonas reinhardtii* flagella harbour two AKAPs; viz., the radial spoke AKAP97 or RSP3 and the central pair AKAP240. Both these were identified on the basis of their RII-binding property. Interestingly, AKAP97 binds *in vivo* to two RII-like proteins (RSP7 and RSP11) that contain only the D/D domain.

**Results:**

We found a *Chlamydomonas* Flagellar Associated Protein (FAP174) orthologous to MYCBP-1, a protein that binds to organellar AKAPs and Myc onco-protein. An *in silico* analysis shows that the N-terminus of FAP174 is similar to those RII domain-containing proteins that have binding affinities to AKAPs. Binding of FAP174 was tested with the AKAP97/RSP3 using *in vitro* pull down assays; however, this binding was rather poor with AKAP97/RSP3. Antibodies were generated against FAP174 and the cellular localization was studied using Western blotting and immunoflourescence in wild type and various flagella mutants. We show that FAP174 localises to the central pair of the axoneme. Using overlay assays we show that FAP174 binds AKAP240 previously identified in the C2 portion of the central pair apparatus.

**Conclusion:**

It appears that the flagella of *Chlamydomonas reinhardtii* contain proteins that bind to AKAPs and except for the D/D domain, lack the conventional a.a. stretches of PKA regulatory subunits (RSP7 and RSP11). We add FAP174 to this growing list.

**Electronic supplementary material:**

The online version of this article (doi:10.1186/s12860-016-0103-y) contains supplementary material, which is available to authorized users.

## Background

Motile cilia/flagella propel eukaryotic cells in aqueous environment or circulate surrounding fluid. The movement is generated by a microtubule-based biological machine, the axoneme. Most axonemes adopt a ‘9 + 2’ format with 9 outer microtubule doublets encircling two microtubule singlets. The former associate with outer dynein arms (ODA), inner dynein arms (IDA) and radial spokes (RS), while the latter and a number of projections constitute the Central Pair (CP) apparatus. These structures along with other less evident complexes operate in concert to generate the rhythmic beating. And, cyclic adenosine monophosphate (cAMP) is a key 2nd messenger that regulates the movement. Studies with isolated axonemes of various mutants of the ODA, IDA, RS, CP and pharmacological inhibitors have implicated cAMP-dependent protein kinase (PKA) and other phosphoenzymes in the dynein-driven microtubule sliding [1–4]. Since isolated axonemes - without flagellar membrane and soluble contents - were used in the sliding assay, it was proposed that a network of phosphoenzymes anchored to the axoneme regulate dynein–driven motility [3–6].

PKA, a holoenzyme of two regulatory and two catalytic subunits, is anchored to scaffold protein; namely, the A-Kinase Anchoring Protein (AKAP). In 1982, AKAPs were first discovered as high affinity binding partners of the regulatory subunit of PKA [7]. Many more were discovered subsequently from several organisms and cell types by an overlay assay using the RII subunit [8]. In general, AKAPs are scaffold proteins sharing little sequence homology, but usually contain three common features – a region for targeting it to a particular micro-compartment, an Amphipathic helix (AH) that binds to a hydrophobic cleft of the Dimerization/Docking domain (D/D) present on the PKA regulatory subunits, and additional motifs that recruit an array of molecules involved in signalling such as other protein kinases, phosphatases, phosphodiesterases, GSK3ß, and small GTPases [9]. This AH which binds to the D/D domain of the regulatory subunit of PKA and non-PKA proteins serves as a parameter for a protein to be designated as AKAP. Recently, an *in silico* approach was adopted to determine amphipathic helices containing proteins which could be candidate AKAPs [10].

Consistent with multiple implicated roles of PKA in ciliated cells, independent studies used RII overlays to reveal a number of AKAPs in this organelle, at least 7 AKAPs in the fibrous sheath surrounding the 9 + 2 axoneme in mammalian sperms [11], one in cilia of the human respiratory tract [12] and two (AKAP97 and AKAP240) in the axoneme of *Chlamydomonas* flagella [13]. Analysis of flagellar mutants lacking specific axonemal complexes showed that AKAP97 is RSP3 in the RS complex, whereas AKAP240 resides in the CP. While this finding is consistent with the role of RS and the CP in regulating dynein motors, RSs isolated from *Chlamydomonas* flagella did not contain any PKA catalytic subunits [14]. Nonetheless, RSP3 and RS indeed harbour features related to PKA and AKAPs. The N-terminus of RSP3 anchors the RS to particular sites in the axoneme. Secondly, RSP3 forms a homodimer [15], each monomer containing an AH for interacting with RSP7 or RSP11 [16] that contains a RII domain but lack any features required for cAMP signalling or phosphorylation [17, 18]. Therefore, the RS in *Chlamydomonas* flagella appears to utilize PKA anchoring mechanism to tether different molecular modules for the function of the RS.

Notably, a number of proteins with a RII domain have been discovered in mammalian cilia and flagella [18]. In addition, accumulated evidence indicates that RII harbours the D/D domain. In fact, two conserved RS proteins contain what is known as the DPY30 domain that share a similar secondary and tertiary structure with the RII domain and bind amphipathic helices of AKAPs [16, 19, 20]. Another AKAP interactor, viz. Myc-binding protein-1 (MYCBP-1) was found to bind to the AH. MYC and MYCBP-1 complex acts as a transcriptional regulator, enhancing the transcription of genes controlled by the E-Box element and leading to erythrocyte differentiation [21, 22]. It was proposed that MYCBP-1, PKA and AKAP95 form a ternary complex in the nucleus negatively regulating the kinase activity [23]. MYCBP-1 operates outside the nucleus as well, especially during the interphase. It was shown that MYCBP-1 interacts with a few AKAPs, such as AKAP149 in sperm mitochondria, its splice variant S-AKAP84 [24, 25], and BIG2, an AKAP in the trans-Golgi network [26]. Here, we show that FAP174 in *Chlamydomonas* flagella behaves like MYCBP-1 in associating with an AKAP, viz. AKAP240 in the C2 microtubule.

## Results

### FAP174 in *Chlamydomonas* is an MYCBP-1 homologue predicted to form a RII-like domain at the N-terminus

**Fig. 1:**
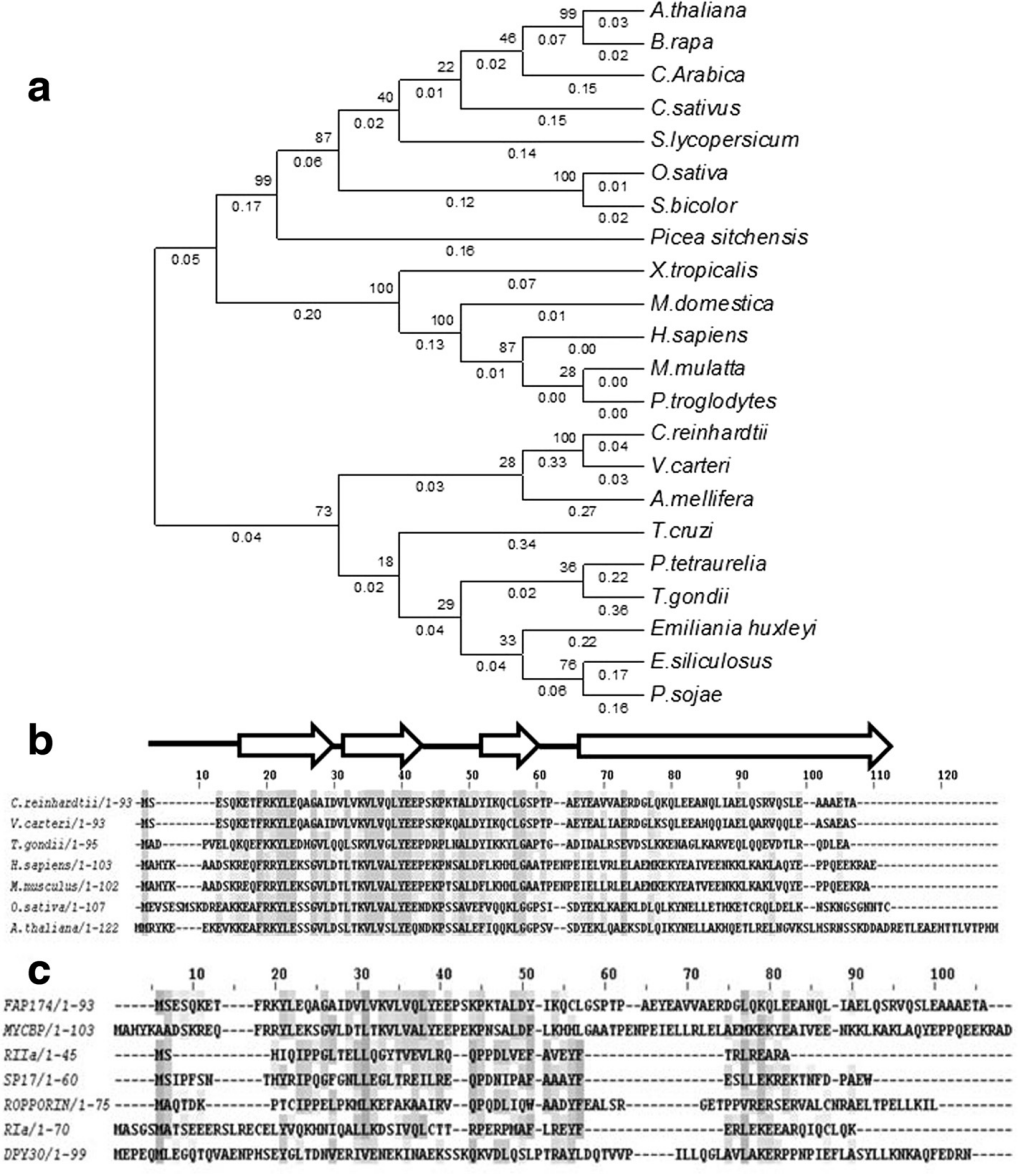
FAP174 harbours a RII-like fold (**a**) A BLAST of FAP174 was carried out and representative MYCBP-1-like sequences from species of plants, animals, algae and protozoans were selected and a phylogenetic tree was generated using MEGA6. Organisms and their Accession numbers used for this exercise are *C. reinhardtii* ACR55627, *V. carterii* XP_002950671.1, *B. rapa* NP_001288931.1, *A. thaliana* NP_671849.1, *C. arabica* ADY38785.1, *C. sativus* XP_004172365.1, *O. sativa* EEC81635.1, *S. bicolor* XP_002459454.1, *S. lycopersicon* XP_004236796.1, *P. sitchensis* ABR18049.1, *A. mellifera* XP_624300.1, *S. kowaleskii* XP_002733639.1, *X. tropicalis* NP_001017035.1, *M. Domestica* XP_001364995.1, *P. troglodytes* XP_003949426.1, *M. mulatta* XP_001113156.2, *H. sapiens* BAA09338.1, *P. tetraaurelia* XP_001442915.1, *T. cruzi* XP_805155.1, *P. sojae* XP_009522570.1, *A. anophagefferens* XP_009033259.1, *E. siliculosus* CBN77061.1 and *T. gondii* XP_002370315.1. (**b**) Alignment of representative sequences from those used for the Phylogenetic analysis. The sequence homology is the highest at the N-terminal region that is predicted to fold into a helix-loop-helix structure as expected of dimerization and docking domains. The C-terminus of FAP174 shows a propensity to form coiled-coils. Identical residues are shaded, arrows represent the helix-forming residues and the lines represent coiled-coil forming residues. (**c**) Multiple alignment of FAP174 with proteins contianing the D/D domain using the best matches from HHpred. (http://toolkit.tuebingen.mpg.de/hhpred). The sequences with the RIIa D/D domain and DPY-30 domains that showed significant match were aligned using Multiple Sequence Alignment software (Clustal Omega). Identical regions are shaded

Several studies have shown that MYCBP-1 is an AKAP interactor [24–27]. BLAST search with the human MYCBP-1 revealed a single homologue, FAP174 in the *Chlamydomonas* flagellar proteome and its presence in other non-ciliated organisms such as angiosperms. Phylogenetic analysis with representative MYCBP-1-like proteins from several organisms generated using MEGA6 [28] showed that FAP174 forms a cluster with the proteins from *Volvox* and protozoans (Fig. 1a). It also appears to have branched from the mammalian lineage of MYCBP-1. Sequence alignment using Clustal Omega [29] of FAP174 and MYCBP-1 from a few species of plants, animals, fungi and protozoans revealed substantial sequence identity or similarity (43–87 %) in the N-terminal region (Fig. 1b). This region also shares a.a. of similar properties with proteins containing the D/D domain similar to that of RII and DPY-30 (Fig. 1c). Homology detection and secondary structure analysis revealed high sequence similarity with the N-terminus that spanned the helix-turn-helix fold typical to the RII clan of proteins (Fig. 1c). This was done using HHpred [30]. The sequences with the RIIa D/D domain and DPY-30 domains that showed significant match were aligned using Multiple Sequence Alignment software (Clustal Omega), (Fig. 1c). From secondary structure prediction of FAP174, it appears that C-terminus is a helix with a strong propensity to form a coiled-coil, known for protein-protein interaction. Therefore, we speculate that FAP174 has two molecular modules, one for binding an AH and one for partnering with proteins (Fig. 1b, c).

### Localization of FAP174

**Fig. 2:**
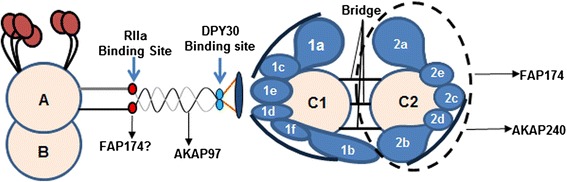
The Radial spoke and Central pair AKAPs. Schematics depicting the two AKAPs in the 9 + 2 axoneme. AKAP97 is RSP3 proposed to be a scaffold protein binding to radial spoke proteins (RSPs) with RII and DPY30 domains. The identity and partners of AKAP240 in C2 of the central pair apparatus remain elusive. This study attempts to identify these interactors. Only part of the 9 + 2 axoneme cross section is illustrated. Also note, that the entire RSP harbours 23 different proteins; however, the figure depicts molecules significant for the current study

**Fig. 3:**
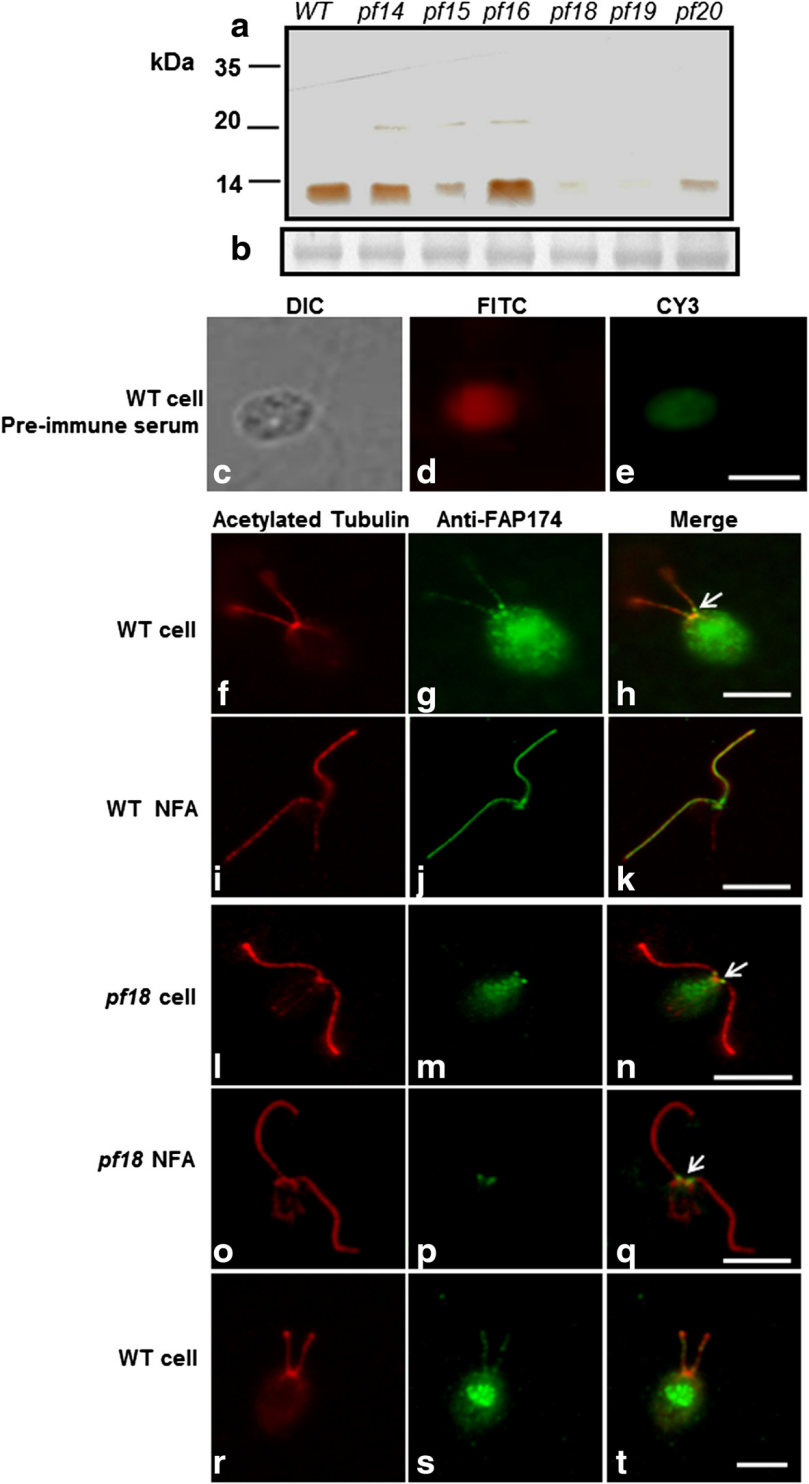
Sub-flagellar localization of FAP174. (**a**) FAP174 Western blot of axonemes from WT and flagellar mutants. A polypeptide of ~10 kDa was present in the axonemes of WT and the radial spokes mutant *pf14*; whereas, among the central pair mutants it was reduced in the *pf15 and pf20, and* absent in *pf18* and *pf19* that lack the central pair microtubules. The presence of FAP174 in *pf16* axoneme that lack the C1 microtubule suggest that FAP174 associates with the C2 microtubule. (**b**) The Ponceau-stained tubulin bands show equal loading of axonemal proteins. (**c**-**e**) Immunofluorescent microscopy of whole cells using pre-immune serum as a control to show autofluorescence and non-specificity of the antibody (images captured at 63x magnification). (**f**-**q**) Immunofluorescent microscopy of FAP174 comparing whole cells and nucleo-flagella apparatus (NFA) from wild type and *pf18*. The samples were decorated with anti-acetylated tubulin or anti-FAP174 as indicated. FAP174 localizes to the flagella as well as to the base of flagella possibly the transition zone (arrows). FAP174 was not detectable in the *pf18* flagella but appeared as the bright spot at the base of flagella. (**r**-**t**) Localization of FAP174 around nucleus is more prominent in cells with low autofluorescence background and one such representative image is depicted here. The scale bars in the figure indicates 10 μm

The *C. reinhardtii* flagella contain two AKAPs; one localized to the RS and the other to the CP [13], (Fig. 2). To determine whether FAP174 is interacting with the AKAP in the RS or CP, we over-expressed 6His-FAP174 in *E. coli*. The freshly purified recombinant protein migrated as ~12 kDa monomers in SDS-PAGE; after storage at −20 °C, it appeared as monomers and stable dimers. The purified protein was used to raise rabbit anti-6His-FAP174 polyclonal antibody. The affinity-purified antibodies were used for probing flagella of WT and representative mutant strains lacking specific axonemal structures. Western blot analysis of axonemes showed that the antibody recognized a single ~10 kDa protein as expected of native FAP174 in WT axonemes (Fig. 3a,b). The band was present in *pf14* lacking the RS and *pf16* that is deficient in the C1 microtubule, but normal in the C2 of the CP apparatus. However, FAP174 is either absent or less abundant in *pf15, pf18, pf19 and pf20* mutants that lacks the entire CP apparatus or is partially defective (Fig. 3a,b). This suggests that FAP174 is present in the C2 microtubule or the associated projections.

For immunofluorescence imaging, whole cells and the isolated Nucleo-Flagellar-Apparatus (NFA, intact flagella in conjunction with the nucleus and centriolar apparatus) were incubated with affinity-purified anti-6His-FAP174 antibody and mouse anti-acetylated tubulin antibody. As compared to the pre-immune serum control (Fig. 3c-e), both, anti-acetylated tubulin (using FITC filter) and anti-FAP174 (using CY3 filter) decorated the entire flagella, the basal bodies of WT cells and NFA (Fig. 3f-k). Notably, two pairs of spots are particularly prominent at the flagellar base. Consistent with the western blot, anti-6His-FAP174 did not decorate mutant *pf18* flagella lacking the CP; but, still revealed the bright spots at the flagellar base (Fig. 3l-q). Taken together, these observations suggest the localization of FAP174 in the C2 part of the CP as AKAP240. The localization near the basal body and transition zone (TZ) could be the protein being transported to the flagella. In cells with less intense autofluorescence from plastids, FAP174 was also evident around the nucleus (Fig. 3r-t ) as reported previously for MYCBP-1 [21].

### *In vitro* interaction of FAP174 with the flagellar AKAPs

**Fig. 4:**
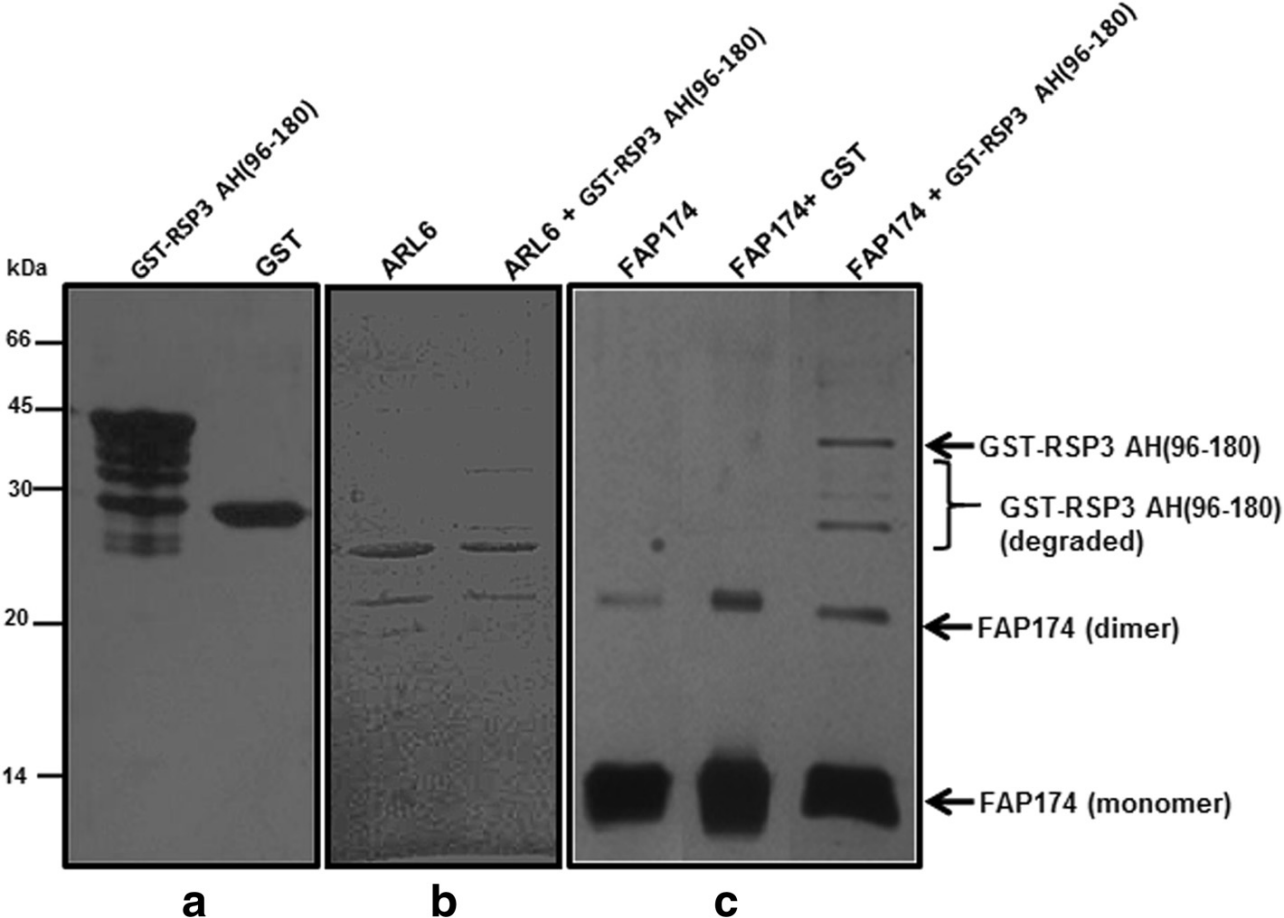
Interaction of FAP174 with GST-RSP3-AH 96–180 *in vitro*. (**a**) Purified proteins used for the assay. (**b**)  Negative control for the assay, which depicts no interaction between Arl6 and GST-RSP3 AH(96-180). (**c**) Co-purification of FAP174 with GST or GST-RSP3-AH 96–180. Testing the *in vitro* interaction of the GST-RSP3-AH 96–180 with 6His-FAP174 protein. Note the rather weak interaction as evidenced in the last lane (arrows)

We tested whether FAP174, like RII, can bind to flagellar AKAPs in overlay and *in vitro* 6His pull-down assays. The recombinant 6His-FAP174 and GST-tagged RSP3 were co-expressed in *E. coli* BL21 (DE3) (arrowheads, Additional file 1: Figure S1). As a control, a clone co-expressing GST with FAP174 was generated. It was found that 6His-FAP174 pulled down GST-RSP3 and another polypeptide (though faint) of a similar molecular weight that was also seen in the GST tag control (Additional file 1: Figure S1). This suggested that the full-length GST-RSP3 did not interact with FAP174 under the current assay conditions. This led us to test the interaction of FAP174 with the RII-Binding AH in RSP3. We generated bacterial strains co-expressing 6His-FAP174 and the AH (spanning 96–180 a.a.) of RSP3, henceforth referred to as GST-RSP3 AH(96–180). Since low co-expression hindered the experiment, individual fusion proteins were purified (Fig. 4a). 6His-FAP174 pulled down the GST-RSP3 AH(96–180) with a low stoichiometry; but, not the GST tag (Fig. 4c). As a control, we tested the interaction between GST-RSP3 AH(96–180) and a non-relevant 6His-tagged recombinant protein (i.e. Arl6) from *Chlamydomonas reinhardtii* (Fig. 4b). With no interaction observed for the negative control (Arl6), we found that FAP174 has rather weak affinity to the AH in RSP3.

**Fig. 5:**
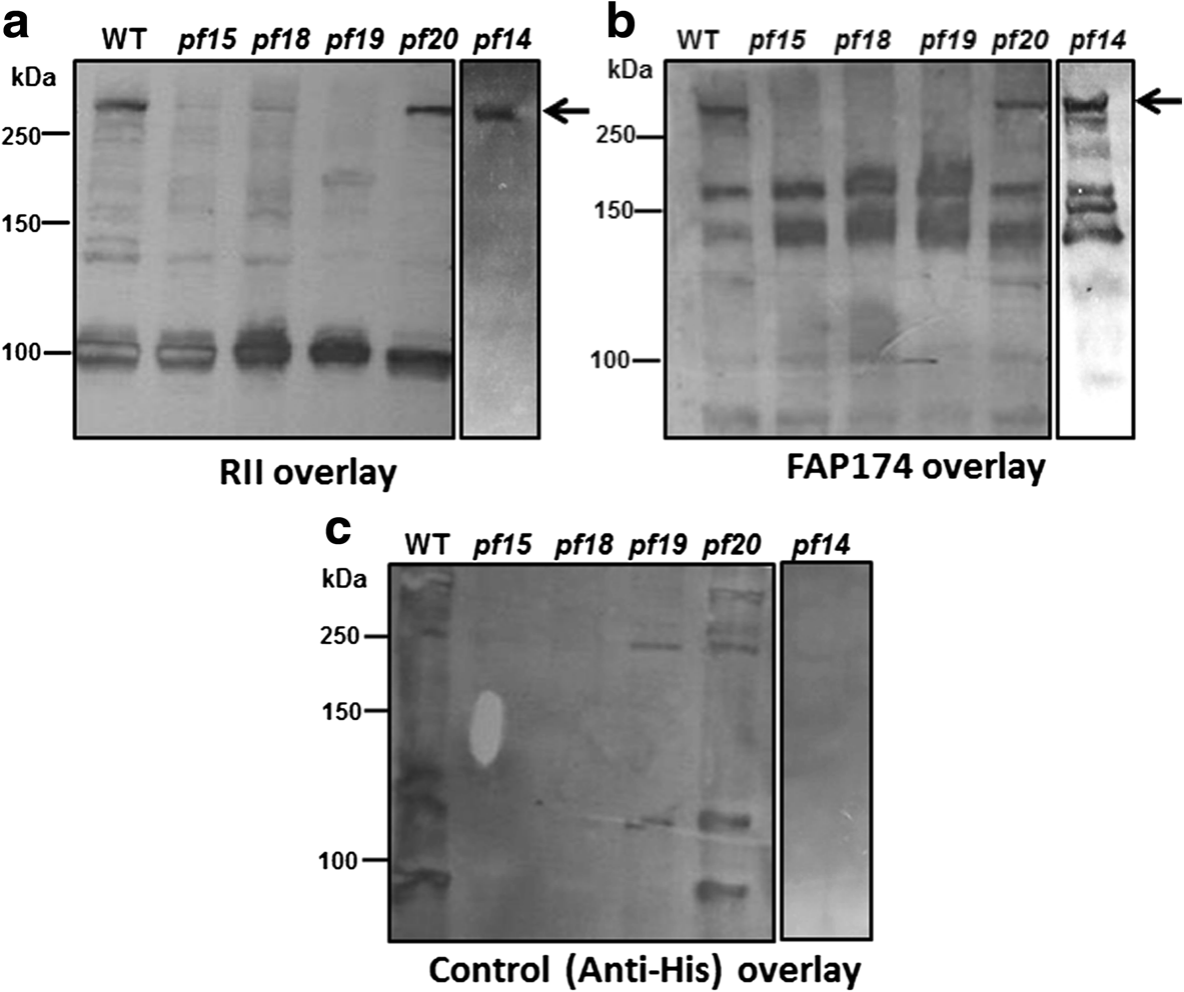
The recognition of AKAP240 by recombinant FAP174 and RII *in vitro*. (**a**) The blots were probed with recombinant 6His-tagged RIIa D/D protein and revealed by HRP-conjugated anti-His antibody. The polypeptide migrated above 250 kDa in the axonemes of WT, *pf14* and *pf20* but were absent in mutants lacking the central pair, as expected of AKAP240. (**b**) 6His-FAP174 overlays also show the same band above 250 kDa. 6His-FAP174 also reacted with two other proteins. The polypeptide migrated above 250 kDa in the axonemes of WT, *pf14* and *pf20* but were absent in mutants lacking the central pair. (**c**) Control overlay blots with WT and mutant axonemes were probed with the anti-His antibody coupled to HRP without the interacting proteins

It may be emphasized that an AKAP240 pull-down assay was not possible, since the AKAP240 gene and protein have not yet been identified. Therefore, we used an overlay assay to test the interaction of 6His-FAP174 and AKAP240 in the axonemes from WT, RS and CP mutants (*pf14, pf15*, *pf18*, *pf1*9 and *pf20*). As compared with the only anti-His antibody control (Fig. 5a), RII overlays on axonemal blots revealed a band corresponding to AKAP240 in the WT, *pf14* and partially defective *pf20* CP mutant (Fig. 5b). In our gels, AKAP240 migrated near 260 kDa. This polypeptide was clearly absent in *pf15, pf18* and *pf19* axonemes that lack the entire CP apparatus. On the other hand, the band corresponding to RSP3 was seen in all the axonemes; except *pf14*. Like RII proteins, 6His-FAP174 in the overlay (Fig. 5c) binds to the expected ~240 kDa molecule in only WT and *pf20* axonemes. Interestingly, though, unlike RIIa (D/D), 6His-FAP174 did not evidently bind to RSP3 in the overlay, consistent with the low stoichiometry co-purification of FAP174 and RSP3 (Fig. 5b,c). The additional two unknown polypeptides of ~150 and 125 kDa whose identity remains elusive were also detected in 6His-FAP174 overlay (Fig. 5c).

## Discussion

MYCBP-1 was found to bind AKAPs in a few organelles, including the AKAPs in flagellar structures that are unique to sperms [21, 22, 26]. The current work demonstrates that the MYCBP-1 orthologue, FAP174, binds to AKAP240 in the axoneme of *Chlamydomonas* flagella. The findings expand the roles of RII-like domain in protein complex assembly and reveal new insight in the composition of the CP apparatus.

Independent lines of evidence indicate that FAP174 is a conserved structural component in a novel molecular complex in the C2 part of CP apparatus. It was first identified in *Chlamydomonas* flagella proteome project [31]. Western blots using the FAP174 specific antibodies showed that it is absent in mutant axonemes missing the C2 microtubule of the CP (Fig. 3a); but, is retained in the mutant axonemes missing the C1 fraction (Fig. 3a). While *pf15* mutant which also shows reduced amounts of FAP174 is considered CP minus, its axoneme often contains an electron-dense core in the place of the CP [32]. It has been demonstrated recently that selective CP proteins remain in the core of CP mutants [33, 34]. The absence of RS, on the other hand, does not affect the assembly of FAP174 (Fig. 3a). Consistent with this, immunofluorescence microscopy show anti-FAP174 decorated the entire flagella of WT but not a CP mutant. Likewise, overlays showed that FAP174 and RIIa (D/D) recognize a 260 kDa polypeptide in the axonemes of WT corresponding to AKAP240 present in the C2 microtubule and no band corresponding to the 97 kDa RSP3 polypeptide. The latter result and its localization only to the central pair suggest that FAP174 is not a physiological interactor of RSP3.

MYCBP-1 was initially identified in the nucleus and the surrounding membranous networks for assisting c-MYC trafficking between the compartments. It also forms a ternary complex with AKAPs and MYCBPAP in the nucleus. It seems that MYCBP-1 uses its RII-like domain to associate with AKAPs and its coiled-coil region to bind c-MYC and MYCBPAP. As far as the presence of FAP174 in basal body or TZ is concerned, the fluorescent signal could be indication of protein carried to these locations. Another interesting scenario could be there are additional AKAPs in the TZ or basal bodies anchoring FAP174 and are yet to be determined. Consistent with this notion, AKAP450 is present in the proteome of the human and fly centrosome [35, 36]. In this regard, FAP174 is rather versatile as proteins that contain the D/D domain (RII clan) is involved in the assembly of several molecular complexes in distinct cellular compartments or organisms. To this extent, it would be very interesting to know the exact sub-flagellar localization of FAP174-AKAP240 complex and the molecular identity of AKAP240.

## Conclusions

The current study has identified a MYCBP-1 like protein with D/D like domain localized in the central pair of the *Chlamydomonas reinhardtii* flagella. Similar to its mammalian equivalent, the D/D domain facilitates its binding to AKAPs. The study further adds FAP174 as a new protein to the C2 microtubule protein complexes. Our further efforts are to characterize this AKAP-FAP174 complex, find the molecular identity of AKAP240 and explore the role of this complex in the motility of the 9 + 2 cilia.

## Methods

The biochemical reagents and media components were obtained from Amresco (USA), SRL (India) and Merck Millipore (India). Ni-NTA Agarose used for protein purification was obtained from Qiagen (Germany). The primers for PCR were obtained from Merck Millipore (Genei, India). Also see Table 1 for list of clones used in the current study.

**Table 1:** List of genes and proteins used/generated in the current study

No.	Insert	Vector	Protein product	Comment	Ref
1	*fap174*	pET28a	6His FAP174	c-Myc Binding protein ortholog	Current study
2	*RSP3*	pGEX2T	GST-RSP3	Radial spoke AKAP full length	[17]
3	*Δrsp3 (96–180)*	pGEX2T	GST-RSP3-AH 96-180	Truncated RSP-3 with Amphipathic helix (96–180 a.a.) that binds to proteins with Dimerization and Docking domains	[14]
4	*arl6*	pET28a	ARL6	Full length GTP-binding protein	From Jacinta D’Souza laboratory
5	*rIIa D/D*	pET15b	RIIa D/D	Regulatory subunit of PKA, truncated protein (1–44 a.a.) with Dimerization and Docking domain	Gift from Susan Taylor laboratory [39]

### *Chlamydomonas reinhardtii* cultures

The wild type (cc124) and mutant strains deficient in the RS (*pf14)* and CP (*pf14*, *pf15*, *pf16*, *pf18*, *pf19,* and *pf20)* and cc620, cc621 were procured from *Chlamydomonas* Resource Center (University of Minnesota). All cells were grown in liquid TAP medium with continuous light under shaking conditions at 25 °C [37].

### Cloning of *fap174* gene, induction, over-expression and purification of FAP174 and other proteins

Primers were designed specific to *fap174* gene with NdeI and EcoRI as flanking restriction sites (FAP174 Nde FP: CATATGATGTCGGAGTCG and FAP174 Eco RP: GAATTCTTATGCAGTCTCCGC). Using the *C. reinhardtii* S1D2 cDNA library as template in a PCR reaction, the amplicons so obtained were cloned into the Nde1 and EcoR1 sites of the pET28a vector and the resultant construct transformed into competent *E. coli* BL21(DE3) cells (GenBank Accession no. FJ986299). The 6His-tagged FAP174 recombinant protein was induced using IPTG. The protein was purified using Ni-NTA Chromatography with minor modifications to the protocol [38]. *Chlamydomonas* RSP-3 and the nucleotide region containing RII-binding AH spanning 96–180 a.a. were cloned in pGEX-2 T vector. Both fusion proteins were induced using 1 mM IPTG and purified using Glutathione-Sepharose 4B (GE Healthcare). Rat RIIa D/D (coding for 1–44 a.a.) cloned in pET15b vector was a kind gift of Dr. Susan Taylor (UCSD, USA). The protein was purified according to procedures described previously [39].

### Extraction of flagella, axonemes and nucleo-flagella apparatus

*Chlamydomonas* cells were grown till mid-log phase and harvested by centrifugation (1,100 *g*/5 mins). The flagella were extracted according to standard protocol [40]. For axoneme extraction, the flagella were de-membranated using 0.5 % IGEPAL CA0630 (Sigma) in HMDEK buffer followed by centrifugation (16,000 *g*/20 mins.). For preparing the Nucleo-Flagella-Apparatus (NFA), [41], autolysin-treated cells were loaded on glass slides and treated with 1X Microtubule stabilising buffer (MT, 30 mM Tris-Cl, 5 mM MgSO_4_, 5 mM EGTA, 25 mM KC1, 1 mM dithiothreitol, pH 7.3) containing 1 % IGEPAL CA0630. Later, the sample on the coverslips was fixed with 1X MT buffer containing 2 % paraformaldehyde followed by washing once with 1X MT and immersing in methanol at −20 °C for 5 min. Later, the NFA’s were used in the indirect immunofluorescence studies.

### Antibody production and indirect immunofluorescence

Purified 6His-tagged FAP174 protein was used as an antigen to immunize rabbits. The antibody generation and affinity purification of anti-FAP174 antibodies was done as a service by Merck-Millipore (Genei, India). These affinity-purified antibodies were used for immunofluorescence assays. For indirect immunofluorescence assay, cc124 cell suspensions were applied to polyethylenimine (PEI)-treated coverslips. Cells were allowed to adhere and subsequently washed to remove those that have not adhered. The coverslip was immersed in methanol at −20 °C for 5 min. The air-dried coverslips were washed and treated with blocking solution (3 % BSA in Phosphate-Buffered Saline, PBS) and then rabbit anti-6His-FAP174 antibody (1:1500 dilution) and mouse anti-acetylated alpha tubulin antibody (1:500 dilution Abcam, 6-11B-1 clone) were added at the same time for 1 h followed by washing with PBST (PBS + 0.05 % Tween-20). Then, incubation with anti-mouse Alexa flour-568 and anti-rabbit Alexa flour-488 (1:100) (Molecular probes, USA) was carried out for 1 h. After washes, coverslips were mounted with a drop of Prolonged^®^ Gold Anti-fade reagent (Molecular Probes, USA) on glass slides and observed using Nikon 90i microscope using plan Flour 63x/1.25 oil immersion objective and appropriate filters. When the primary antibody was replaced with a pre-immune antibody and the entire procedure repeated as above, these cells served as a control.

### ECL and overlay assays

Purified protein and flagellar/axonemal extracts were electrophoresed on SDS-PAGE gels and transferred onto a nitrocellulose membrane. The membrane was stained for the presence of proteins with Ponceau S and subsequently blocked with 3 % BSA in TBS + 0.05 % Tween-20 (TBST). For overlay assay, the membrane was overlaid with ~20 μg/ml purified FAP174 or RIIa (D/D) protein after blocking. The control blot (anti-His only) was not overlaid with any protein with the subsequent steps same as that of the test blot. Subsequent steps in both the cases involved washing 3 times with 1 % BSA in TBST and probing with primary and secondary antibodies at appropriate dilutions. The membranes were developed using ECL-Advance (GE Healthcare, USA) and the signal was revealed using conventional X-Ray plates, developers and fixers.

### Protein-protein interaction assays (Co-expression, His-Pull-down assay)

For co-expression and pull-down studies, modified procedure of Yang and Yang (2006) was followed. Briefly, plasmids harbouring different genes [*fap174*, *rsp3* and *∆rsp3(96–180)*] and selection antibiotics were co-transformed into *E. coli* BL21(DE3) cells and the proteins over-expressed after induction with 1 mM IPTG. As a control, empty pGEX2T plasmid was co-transformed with the *fap174* construct. Upon appropriate selection, positive clones were used to pull-down the 6His-FAP174 protein with Ni-NTA resin. Pull-down assays were carried out using respective lysates from co-transformants or pure proteins (FAP174, RSP3 or GST-RSP3-AH 96–180) or GST as a control). For His-pull-down assays, the cell lysates or purified proteins were incubated with the Ni-NTA resin at 4 °C for 1 h, followed by three washes with assay buffer (25 mM Tris-Cl, pH 7.5, 150 mM NaCl, 5 % Sucrose, 0.1 % Triton-X, 0.1 mM PMSF, 50 mM Imidazole). The interactor was then added and the mixture incubated at 4 °C for 2 h, followed by three washes with the same buffer. Subsequent electrophoresis and silver staining revealed the interactors.

## Abbreviations

AH, amphipathic helix; AKAP, A-Kinase anchoring protein; ARL6, ADP ribosylation factor-like 6 protein; BIG2, brefeldin a-inhibited guanine nucleotide-exchange factor 2; BLAST, basic local alignment search tool; BSA, bovine serum albumin; cAMP, cyclic adenosine monophosphate; c-Myc, myelocytomatosis; CP, central pair; D/D domain, dimerization docking domain; DPY-30, Dumpy-30; ECL- Enhanced chemiluminesence; FAP174, flagella associated protein 174; GSK3β, glycogen synthase kinase 3beta; GST, glutathione-S-transferase; IDA, inner dynein arms; IPTG, isopropyl β-D-1-thiogalactopyranoside; kDa, kilo Dalton; MEGA6, molecular evolutionary genetics analysis version6; MT buffer, microtubule stabilising buffer; MYCBP-1, MYCBinding protein-1; MYCBPAP, Myc-binding protein associated protein; NFA, nucleo-flagellar apparatus; Ni-NTA, nickel-nitrilotriacetic acid; ODA, outer dynein arms; PBS, phosphate buffered saline; PCR, polymerase chain reaction; PEI, polyethyleneimine; PKA, cAMP-dependent protein kinase; PMSF, phenylmethylsulphonyl fluoride; RS, radial spoke; RSP, radial spoke protein; SDS-PAGE, sodium dodecyl sulphate-polyacrylamide gel electrophoresis; TBS, Tris buffered saline; TZ, transition zone; WT, Wild type

## Additional file

Additional file 1: Figure S1. Testing the interaction of FAP174 with GST-tagged RSP3 *in vitro*. (A) Co-expression of FAP174 with GST or GST-tagged RSP3. The first, second and fourth lanes are the crude lysates with independently expressing FAP174, GST and GST-RSP3 proteins (see arrowheads); the third lane is the clone over-expressing both the FAP174 and GST proteins; while, the last lane is the lysate from the clone over-expressing both FAP174 and GST-RSP3 (see arrowheads). (B) FAP174 (arrow at the bottom of the gel) was co-purified with GST-RSP3 (arrow at the top of the gel) but not with GST alone. FT, W and E are Flow-through, Wash and Elutes, respectively. Note that there no interaction between full-length RSP3 and FAP174. (PNG 379 kb)
