# Supplementary figures and images for: Myc-binding protein orthologue interacts with AKAP240 in the central pair apparatus of the Chlamydomonas flagella
Source: BMC Cell Biol. 2016 Jun 10;17:24. doi: 10.1186/s12860-016-0103-y (PMC4901443; doi:10.1186/s12860-016-0103-y)

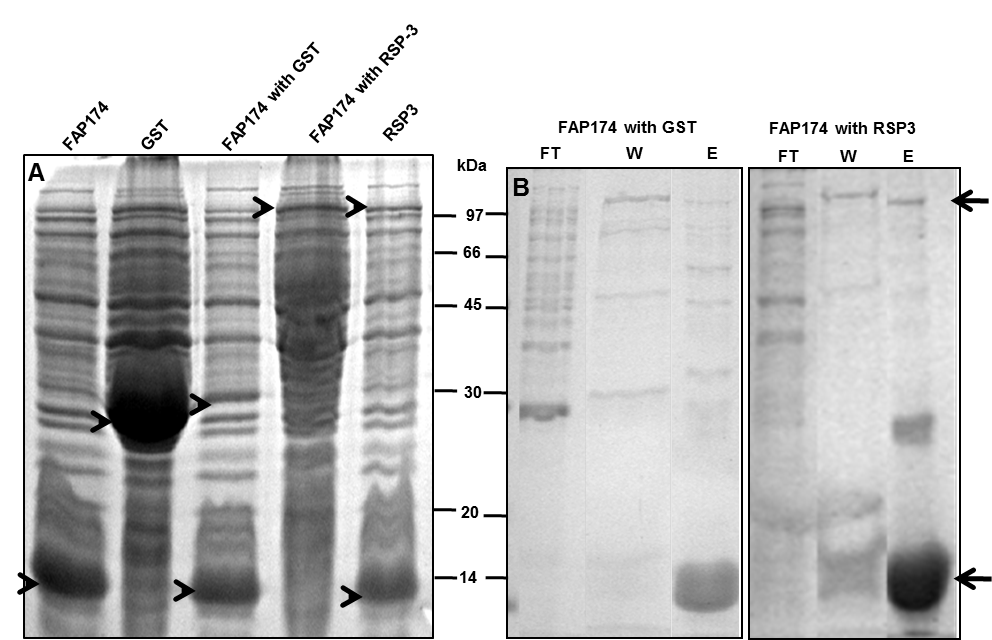

Supplement: Additional file 1: Figure S1. — Testing the interaction of FAP174 with GST-tagged RSP3 in vitro. (A) Co-expression of FAP174 with GST or GST-tagged RSP3. The first, second and fourth lanes are the crude lysates with independently expressing FAP174, GST and GST-RSP3 proteins (see arrowheads); the third lane is the clone over-expressing both the FAP174 and GST proteins; while, the last lane is the lysate from the clone over-expressing both FAP174 and GST-RSP3 (see arrowheads). (B) FAP174 (arrow at the bottom of the gel) was co-purified with GST-RSP3 (arrow at the top of the gel) but not with GST alone. FT, W and E are Flow-through, Wash and Elutes, respectively. Note that there no interaction between full-length RSP3 and FAP174. (PNG 379 kb) [file 12860_2016_103_MOESM1_ESM.png]
